# An interpretable multimodal ensemble assessment framework for Alzheimer’s disease cognitive staging

**DOI:** 10.3389/fneur.2026.1911900

**Published:** 2026-08-05

**Authors:** Jiale Zhang, Bo Yuan, Yaran Liu, Zhidong Xue

**Affiliations:** 1 Shandong Key Laboratory of Complex Medical Intelligence and Aging, Shandong Medical and Pharmaceutical University, Yantai, China; 2 Institute of Artificial Intelligence, Beihang University, Beijing, China; 3 Beijing Advanced Innovation Center for Future Blockchain and Privacy Computing, Beihang University, Beijing, China; 4 School of Health Management, Shandong Medical and Pharmaceutical University, Yantai, China; 5 School of Software Engineering, Huazhong University of Science and Technology, Wuhan, China

**Keywords:** Alzheimer’s disease, artificial intelligence, cognitive staging, interpretability, multimodal learning

## Abstract

**Objectives:**

Alzheimer’s disease (AD) is the most prevalent type of neuro-degenerative dementia. Artificial intelligence assisted clinical evaluation can improve diagnostic efficiency and facilitate timely intervention.

**Methods:**

An interpretable Multimodal Ensemble Assessment Framework (MEAF) was developed to support clinical evaluation for AD across the cognitive spectrum. This framework employed a Swin Transformer and ResNet-50 for staged modeling of imaging features, applied machine learning techniques to extract clinical features, and used decision-level ensemble learning to integrate multimodal predictions. For interpretability analysis, Gradient-weighted Class Activation Maps were used to highlight key brain regions contributing to imaging-based decisions, and Shapley Additive exPlanations were applied to quantitatively assess the importance of clinical features.

**Results:**

MEAF achieved robust performance in classifying cognitively normal, mild cognitive impairment, and AD, with an accuracy of 0.878 and an F1-score of 0.877. In the independent external validation cohort, MEAF maintained reasonable performance, with an accuracy of 0.817 and an F1-score of 0.803. Interpretability analyses provided complementary explanations for both the imaging and clinical models.

**Conclusion:**

MEAF demonstrated favorable classification performance and interpretability in retrospective multicenter datasets, suggesting its potential as an auxiliary framework for multimodal assessment of AD-related cognitive staging.

## 1 Introduction

Alzheimer’s disease (AD) is a progressive neurodegenerative disorder characterized by the gradual decline in memory, cognition, and behavior (1). It is the leading cause of dementia worldwide and represents a major and growing global public health challenge (2). By 2050, the number of individuals living with dementia worldwide is projected to reach approximately 153 million (3). Currently, no curative therapy is available for AD, and existing treatments are limited to delaying disease progression (4, 5). Due to the incomplete understanding of AD pathophysiology and the nonspecific presentation of early symptoms, accurate diagnosis at the prodromal stage remains challenging, often leading to delays in intervention (6, 7). Therefore, the development of accurate and interpretable diagnostic tools is critical for early disease detection, timely intervention, and the improvement of clinical outcomes (8, 9).

Currently, the clinical diagnosis of Alzheimer’s disease relies primarily on conventional diagnostic assessments, including medical history review, neurocognitive testing (10, 11), magnetic resonance imaging (MRI) (12, 13), and biomarker analyses, such as genetic and protein markers (2, 14). However, these approaches are limited by their reliance on expert interpretation and by their low sensitivity to early pathological changes, particularly when subtle abnormalities, such as mild hippocampal atrophy, are difficult to detect, thereby increasing the risk of misdiagnosis or underdiagnosis (15, 16).

To address these limitations, artificial intelligence (AI)-based models have been increasingly employed in AD research, facilitating the automated analysis of diverse biomedical data and enhancing diagnostic efficiency and accuracy (17). Deep learning and other data-driven methods trained on single-modality data have demonstrated promising performance in AD classification (18–22). However, exclusive reliance on single-modality data fails to capture the complex and heterogeneous nature of AD pathology, thereby limiting the generalizability and clinical utility of such models (23, 24). With the growing availability of biomedical data, recent advances in multimodal fusion have demonstrated considerable potential for addressing these challenges (25, 26). By integrating MRI scans, demographic variables, clinical assessments, and molecular biomarkers, multimodal frameworks provide a more comprehensive representation of disease progression and allow for more accurate differentiation among CN, MCI, and AD (27, 28). For example, dual-model frameworks combining clinical assessment and MRI-based analysis have demonstrated the complementary diagnostic value of these two modalities (29). Cross-modal attention networks further integrate imaging, clinical, and genetic information to support multiclass AD classification (30). In addition, NeuroNet-AD, a recently proposed end-to-end multimodal framework, has shown the effectiveness of combining heterogeneous clinical and neuroimaging information for multiclass AD diagnosis (31). In most existing studies, primary emphasis has been placed on predictive performance, with limited attention devoted to model interpretability (32). Consequently, the internal decision-making processes of these models remain difficult to interpret, particularly in high-risk or safety-critical clinical settings (33). Therefore, interpretability analysis remains a critical challenge in the development of trustworthy AI systems to assist clinical assessment of AD.

This research presents a clinically oriented Multimodal Ensemble Assessment Framework (MEAF) for cognitive staging across the AD continuum. Built upon established imaging and clinical modeling approaches, MEAF was designed to integrate structural MRI and routine clinical variables through a modular decision-level ensemble strategy. In this framework, imaging and clinical data were modeled separately, allowing each modality to contribute complementary information while preserving modality-specific interpretability. The prediction probabilities from the imaging and clinical models were then combined at the final decision stage to support CN/MCI/AD classification. Using retrospective multicenter datasets from ADNI, OASIS, and NACC for model development and internal evaluation, and an independent AIBL cohort for external validation, we evaluated the classification performance of MEAF and conducted subgroup analyses to examine the consistency of model performance across different patient subgroups. In addition, Grad-CAM and SHAP analyses were applied to provide visual and feature-level explanations for imaging- and clinical-model predictions, respectively. Overall, this study focuses on the framework-level integration and evaluation of multimodal information for interpretable assessment of AD-related cognitive stages.

## 2 Materials and methods

### 2.1 Study population

This study employed data from four publicly accessible neuroimaging databases, including the Alzheimer’s Disease Neuroimaging Initiative (ADNI) (34), the Open Access Series of Imaging Studies (OASIS) (35), the National Alzheimer’s Coordinating Center (NACC) (36), and the Australian Imaging, Biomarkers and Lifestyle Flagship Study of Ageing (AIBL) (37). For each participant, the data consisted of age, sex, educational attainment, Mini-Mental State Examination (MMSE) scores, Clinical Dementia Rating (CDR) scores, Geriatric Depression Scale (GDS) scores, Boston Naming Test (BNT) scores, and structural T1-weighted magnetic resonance imaging (T1WI) scans. Note that MMSE and CDR are routine cognitive scales collected in the original multicenter cohorts, and we retain all available clinical indicators to simulate complete real-world clinical assessment data rather than relying solely on pathological imaging biomarkers.

Subsequently, the merged ADNI/OASIS/NACC dataset was divided into training and internal testing subsets in an 8:2 ratio using stratified random sampling to preserve the distribution of diagnostic groups. Each participant was included only once and assigned exclusively to one subset, with the participant-level split completed before model training, hyperparameter tuning, data augmentation, and model selection. No oversampling, undersampling, class weighting, or other class-rebalancing strategy was applied. To reduce the potential impact of class imbalance on model evaluation, accuracy, precision, specificity, F1-score, confusion matrices, and class-wise performance metrics were reported in addition to AUC.

**Figure 1:**
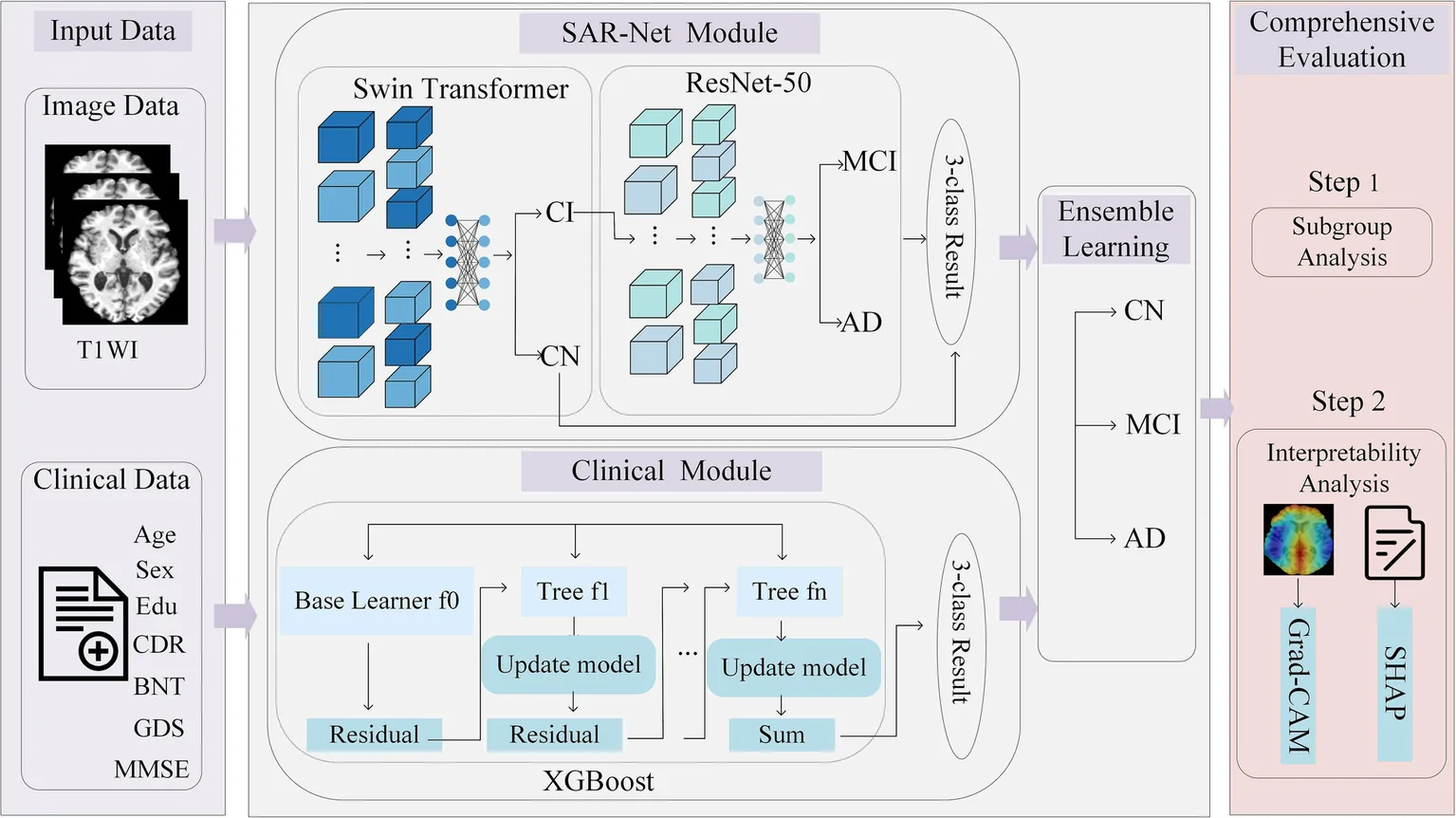
The flow diagram of the whole study.

The random split of the merged datasets was considered an internal testing strategy rather than cohort-independent validation. Therefore, we additionally included the AIBL cohort as an independent external validation cohort, which was kept completely separate from model development and evaluated using the available demographic, cognitive, and T1WI data. Figure 1 illustrates the overall workflow of the study.

### 2.2 MRI image preprocessing

**Figure 2:**
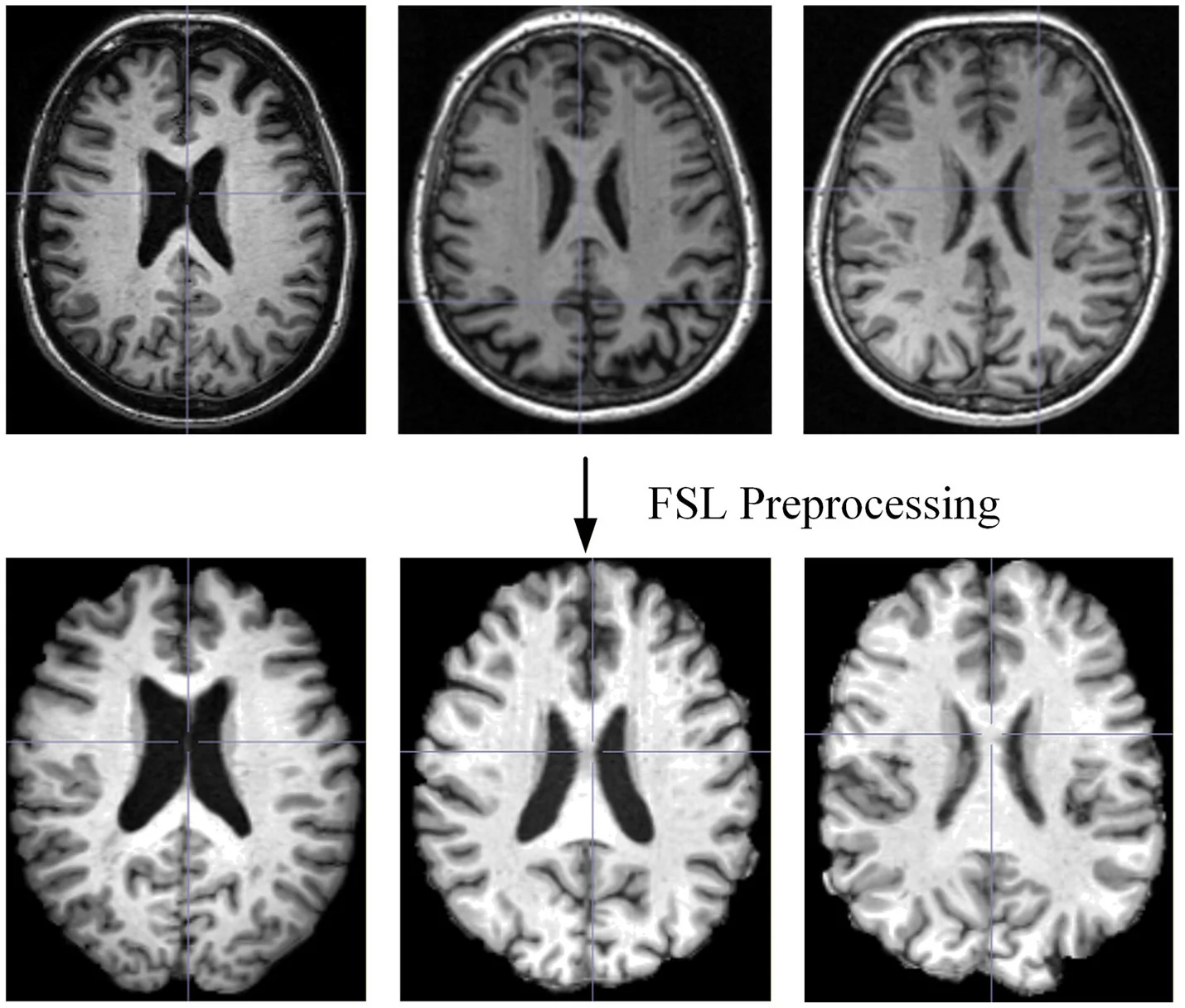
Caption image preprocessing sample. The top row shows the original MRI scans, while the bottom row illustrates the corresponding images following FSL preprocessing.

To mitigate heterogeneity arising from variations in imaging protocols and scanner parameters across multicenter datasets, all MRI images were processed using a standardized preprocessing pipeline. MRI preprocessing was performed independently for each participant using a fixed pipeline without using information from other participants or diagnostic labels. All preprocessing procedures were performed using the FMRIB Software Library (FSL) toolkit (38). First, N4 bias field correction, implemented through ANTs, was applied to correct intensity inhomogeneities induced by magnetic field nonuniformity (39), thereby improving overall image consistency and quality. Skull stripping was subsequently performed using the Brain Extraction Tool (BET) within FSL to exclude non-brain tissues. Next, linear registration was performed using the FLIRT tool within FSL to align each image to the standard MNI space (MNI152 1 mm template) (40). Finally, voxel intensity normalization was applied to enhance training stability and promote model convergence (Figure 2).

### 2.3 Evaluation of MRI preprocessing

To evaluate the effectiveness of MRI preprocessing and identify potential sources of bias associated with cohort, field strength, or scanner characteristics, t-distributed stochastic neighbor embedding (t-SNE) was employed to visualize the intrinsic data distribution (41). Prior to t-SNE visualization, MRI scans were downsampled by a factor of eight using SimpleITK with B-spline interpolation. Z-score normalization was subsequently applied to MRI-derived features, followed by dimensionality reduction using principal component analysis (PCA). The silhouette coefficient was calculated to quantitatively assess cluster compactness and class separability (42). The silhouette coefficient for each sample i is calculated using Equation 1 as follows:


$$S(i)=\frac{b(i)-a(i)}{\max(a(i),b(i))}$$ (1)

where a(i) is the average intra-cluster distance, b(i) is the nearest inter-cluster distance, and S(i) ranges from −1 to 1. In addition, a domain-classifier analysis was performed to quantitatively assess residual domain-related information after preprocessing (43). Logistic regression classifiers with class-balanced weighting were trained using stratified five-fold cross-validation to predict cohort, magnetic field strength, and scanner manufacturer from the PCA-reduced MRI features. If the model performance was close to chance level, this indicated that only weak residual domain-specific information remained in the imaging features.

### 2.4 MEAF architecture and implementation

#### 2.4.1 Development of MRI module

**Figure 3:**
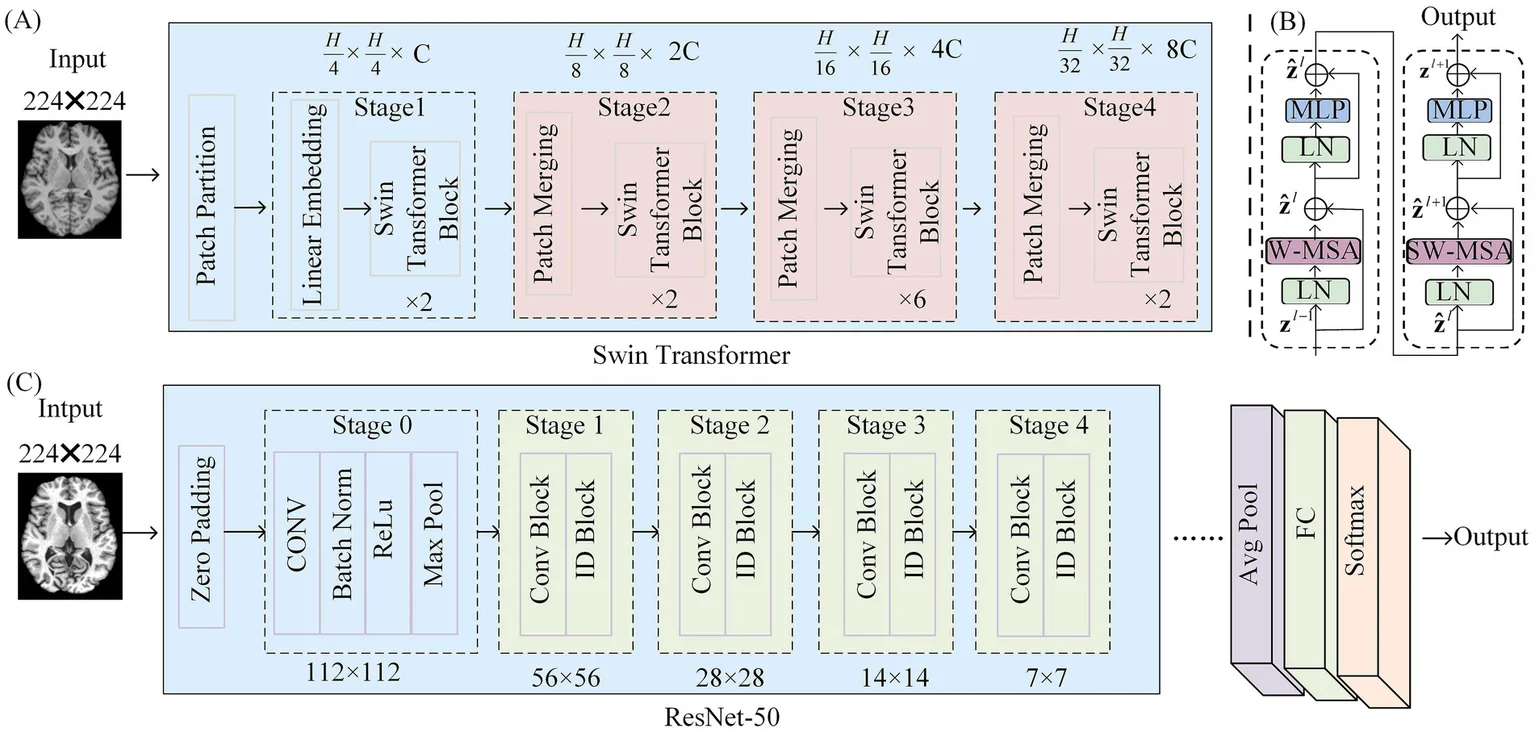
Schematic diagrams of the network architectures. **(A)** The architecture of the Swin Transformer; **(B)** Multiple successive Swin Transformer blocks with multi-head self-attention; **(C)** The architecture of ResNet-50.

To perform MRI-based cognitive classification, a two-stage MRI classification module, termed SAR-Net, was implemented by integrating Swin Transformer and ResNet-50 models within the MEAF framework to more closely mimic the real-world screening workflow. The structures of the individual models are illustrated in Figure 3. The input to the classification network consisted of preprocessed sagittal T1-weighted magnetic resonance imaging (T1WI) slices with a resolution of 224 × 224 pixels. During training, random horizontal flipping with a probability of 0.5 was applied as data augmentation to improve model generalizability.

In the first stage, a Swin Transformer model was employed as the initial classifier to learn feature representations from MRI images for distinguishing CN individuals from Cognitive Impairment (CI) subjects. Benefiting from its hierarchical self-attention mechanism and shifted window strategy, the Swin Transformer is capable of capturing both global and local structural dependencies in MRI images, thereby providing robust feature representations for early cognitive state discrimination (44). The model was trained using the Adam optimizer with a batch size of 4, a learning rate of 0.001, and 100 training epochs. In the second stage, a ResNet-50 network was utilized to further classify CI subjects into MCI and AD (45). The residual learning framework of ResNet-50 facilitates effective gradient propagation and mitigates the vanishing gradient problem, enabling the extraction of fine-grained pathological features associated with disease progression. After deep feature extraction, the feature maps were reduced to fixed-dimensional vectors using an average pooling layer, followed by a fully connected layer to output class probabilities. The model was trained using the Adam optimizer with a batch size of 6, an initial learning rate of 0.0001, and 100 training epochs. The hyperparameter settings of the imaging models were determined based on five-fold cross-validation within the training set, and the testing set was used only for final performance evaluation.

Following the two-stage training process, the proposed framework performs three-class classification of CN, MCI, and AD based on imaging data.

#### 2.4.2 Development of clinical module

Ten mainstream machine learning algorithms were evaluated for diagnostic category prediction using subjects’ clinical characteristics, including logistic regression, artificial neural networks, decision tree models, and various tree algorithms boosted by gradients (46). Their performance was compared using accuracy, precision, recall, F1-score, and AUC. During AIBL external validation, unavailable or non-harmonized clinical variables were retained as missing values and handled according to the missing-value strategy of the final selected clinical model.

#### 2.4.3 Ensemble learning module

To evaluate the effect of fusion strategy selection, we compared several decision-level fusion methods, including equal-weight probability averaging, Borda count-based rank fusion, entropy-based confidence-selection fusion, and stacking-based fusion (47–50). All methods were evaluated under identical experimental settings using the same data partition, modality-specific predictions, and evaluation metrics.

### 2.5 Subgroup analysis

Comprehensive subgroup analyses were conducted to further evaluate the performance of the final integrated model across key demographic variables (age, sex, and educational attainment). Participants were stratified into subgroups as follows: age (≤65 years, 66–80 years, >80 years), sex (male, female), and educational attainment (below high school, high school, university, postgraduate and above).

### 2.6 Interpretability analysis

Explainable AI (XAI) techniques were employed to elucidate the decision-making process of the multimodal framework and to interpret both the imaging and clinical sub-models (51).

Interpretability Analysis of the Imaging Model: The Grad-CAM method was employed to conduct a visual interpretability analysis of the MRI sub-model, with the aim of localizing the anatomical regions most critical for prediction (52). In the preprocessing stage, all MRI images were first resized to a uniform resolution of 224 × 224 to meet the input requirements of the model. Forward and backward hooks were registered on the final feature extraction layer to extract the corresponding feature maps and gradients. For Transformer-based models, the output token sequences were further reconstructed into two-dimensional spatial feature maps. Subsequently, in accordance with the standard Grad-CAM procedure, the channel-wise weights were computed via global average pooling of the gradients, as defined in Equation 2:


$$\alpha_{k}=\frac{1}{Z}\sum_{i}\sum_{j}\frac{\partial y^{c}}{\partial A_{\mathrm{ij}}^{k}}$$ (2)

where $\alpha_{k}$ is the channel weight of the k -th feature map, $y^{c}$ is the predicted score for class c , $A_{\mathrm{ij}}^{k}$ is the activation at spatial location (i,j) of the k -th feature map, and Z is the total number of spatial locations in the feature map. Using these weights, a weighted combination of the feature maps was computed to generate an initial heatmap, which was then processed using ReLU activation and normalization to emphasize regions with high responses. The final Grad-CAM heatmap is defined in Equation 3 as follows:


$$L_{\text{Grad}-\mathrm{CAM}}^{c}=\text{ReLU}(\sum_{k}\alpha_{k}A^{k})$$ (3)

where $L_{\text{Grad}-\mathrm{CAM}}^{c}$ is the Grad-CAM heatmap for class c , $\alpha_{k}$ is the channel weight of the k -th feature map, and $A^{k}$ is the k -th feature map. Finally, the heatmap was upsampled to the original image resolution and overlaid onto the input image using weighted overlay, thereby enabling intuitive visualization of brain regions critical to the model’s predictions.

Interpretability Analysis of the Clinical Model: For the best-performing clinical model in the experiments described above, the SHAP method was employed to elucidate the inner workings of the machine learning model (53). The SHAP explainer was initialized according to the model type: TreeExplainer was employed for tree-based models, and LinearExplainer was employed for linear models. SHAP values were subsequently computed to quantify each feature’s contribution to the model’s predictions. To quantify the stability and predictive relevance of SHAP explanations, we adopted bootstrap resampling to count how often each feature ranked in the top 5 for stability assessment. We further performed SHAP-based feature ablation: removing the top 1/3/5 features or retaining only the top 5 features, then compared AUC and MCC against the full-feature baseline model.

## 3 Results

### 3.1 Participant characteristics

The model development cohort included 1,146 participants, comprising 394 CN, 224 MCI, and 528 AD subjects. The independent AIBL external validation cohort included 186 participants, comprising 44 CN, 55 MCI, and 87 AD subjects. Their demographic and clinical characteristics are summarized in Table 1.

**Table 1:** Demographic and clinical characteristics of subjects.

Variable	Train and internal test (*n* = 1,146)			External validation (*n* = 186)		
	CN (*n* = 394)	MCI (*n* = 224)	AD (*n* = 528)	CN (*n* = 44)	MCI (*n* = 55)	AD (*n* = 87)
Age (Mean ± SD)	72.50 ± 10.64	71.09 ± 8.66	74.50 ± 8.44	72.25 ± 5.76	74.56 ± 7.54	74.47 ± 7.92
Sex Male (*n*, %)	136 (34.5%)	125 (55.8%)	238 (45.1%)	22 (50.0%)	31 (56.4%)	38 (43.7%)
Edu (Mean ± SD)	2.80 ± 0.97	3.12 ± 1.06	2.53 ± 1.12	–	–	–
MMSE (Mean ± SD)	28.49 ± 2.25	27.38 ± 2.18	22.98 ± 4.76	28.8 ± 1.23	26.84 ± 2.36	19.83 ± 6.15
CDR (Mean ± SD)	0.10 ± 0.20	0.46 ± 0.16	0.71 ± 0.37	0.05 ± 0.15	0.47 ± 0.11	0.97 ± 0.59
GDS (Mean ± SD)	1.32 ± 1.58	1.62 ± 1.56	1.22 ± 1.50	–	–	–
BNT (Mean ± SD)	21.95 ± 7.10	24.39 ± 9.19	13.38 ± 3.96	–	–	–

### 3.2 Quantitative assessment of MRI preprocessing

**Figure 4:**
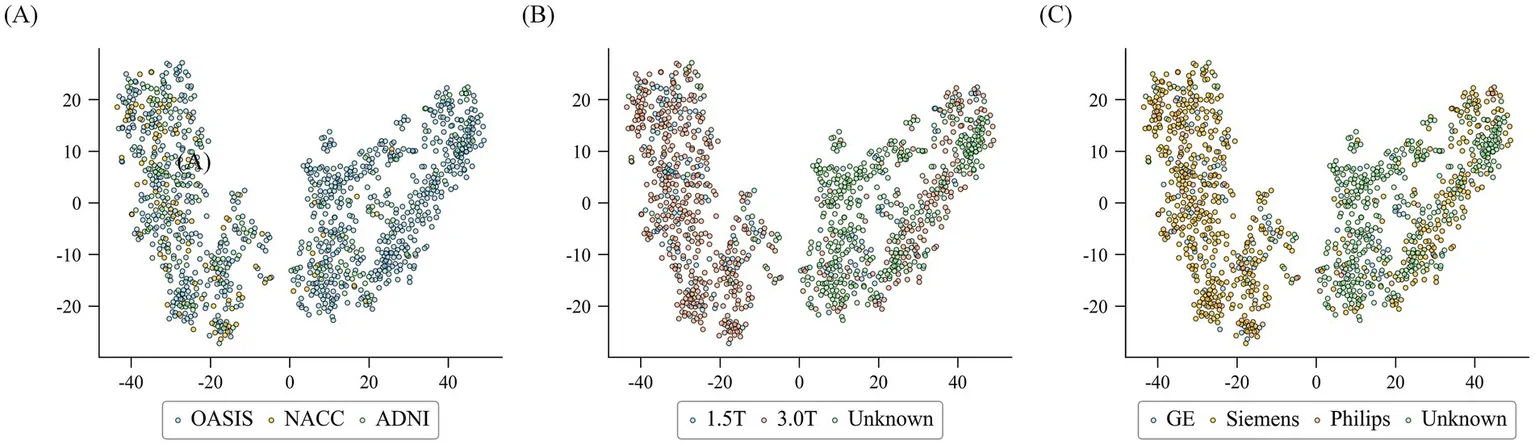
Multimodal data visualization and sample distribution analysis. **(A)** Points colored by cohort; **(B)** Points colored by field strength (1.5 T, 3.0 T), including unknown; **(C)** Points colored by scanner manufacturer, including unknown.

Figure 4 displays three 2D t-SNE visualizations of the preprocessed MRI features. Subplots (A–C) show the distributions stratified by study cohort, magnetic field strength, and scanner type, respectively. No distinct clustering patterns were observed across all three stratifications. The corresponding silhouette coefficients were −0.072, 0.004, and 0.035, all of which were close to zero, suggesting minimal separation in the feature space. Domain-classifier analysis yielded balanced accuracies of 0.398, 0.336, and 0.464 for predicting study cohort, scanner manufacturer, and magnetic field strength, respectively, compared with nominal chance levels of 0.333, 0.250, and 0.333. These results indicated limited but detectable residual domain-related information after preprocessing.

### 3.3 Module classification performances

The predictive performance of the staged MRI-based classification module was robust on the test set. Following integration of both stages, the final MRI model achieved an accuracy of 0.838, indicating robust overall performance (Table 2). Additional evaluation metrics further supported the reliability of the model, with a precision of 0.846, an F1-score of 0.839, a specificity of 0.923, and an AUC of 0.937.

**Table 2:** Performance comparison of MEAF, its component models, and baseline multimodal models.

Model	PRE	F1-score	ACC	SPC	AUC
SAR-Net	0.846	0.839	0.838	0.923	0.937
XGBoost	0.811	0.807	0.808	0.880	0.935
DAFT	0.784	0.782	0.830	0.916	0.830
HyperFusion	0.809	0.810	0.847	0.924	0.866
MEAF	**0.880**	**0.877**	**0.878**	**0.940**	**0.955**
MEAF (external validation)	0.843	0.803	0.817	0.853	0.834

PRE, precision; ACC, accuracy; SPC, specificity; AUC, macro-averaged area under the receiver operating characteristic curve. Bold values indicate the performance of the final integrated MEAF framework on the internal test set.

**Figure 5:**
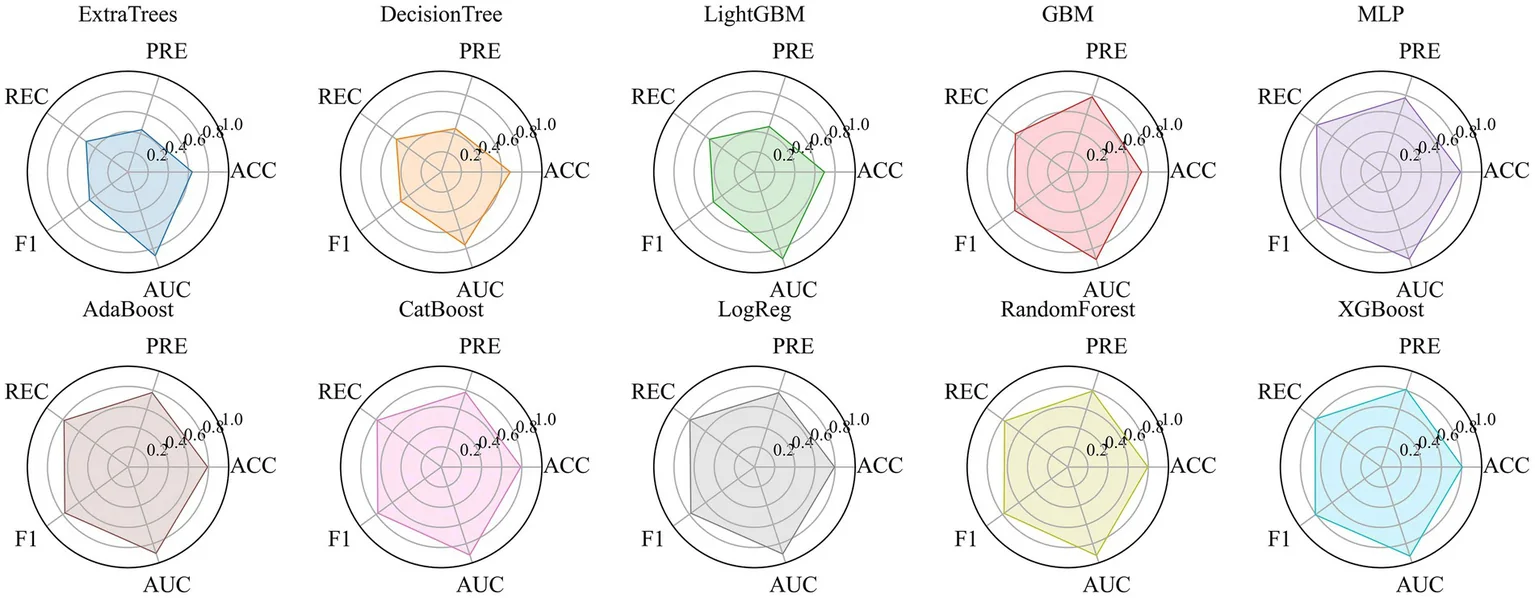
Radar plots summarizing the performance of ten ML algorithms across five evaluation metrics: accuracy (ACC), F1-score (F1), AUC, precision (PRE), and recall (REC).

Ten conventional machine learning algorithms were evaluated on the test set to identify the optimal model for three-class classification of CN, MCI, and AD. The discriminative performance of the models varied, with XGBoost demonstrating the best overall performance, followed by Random Forest (RF) (Figure 5). XGBoost exhibited balanced performance across additional metrics, with a precision of 0.811, an F1-score of 0.807, an accuracy of 0.808, a specificity of 0.880, and an AUC of 0.935 (Table 2).

**Figure 6:**
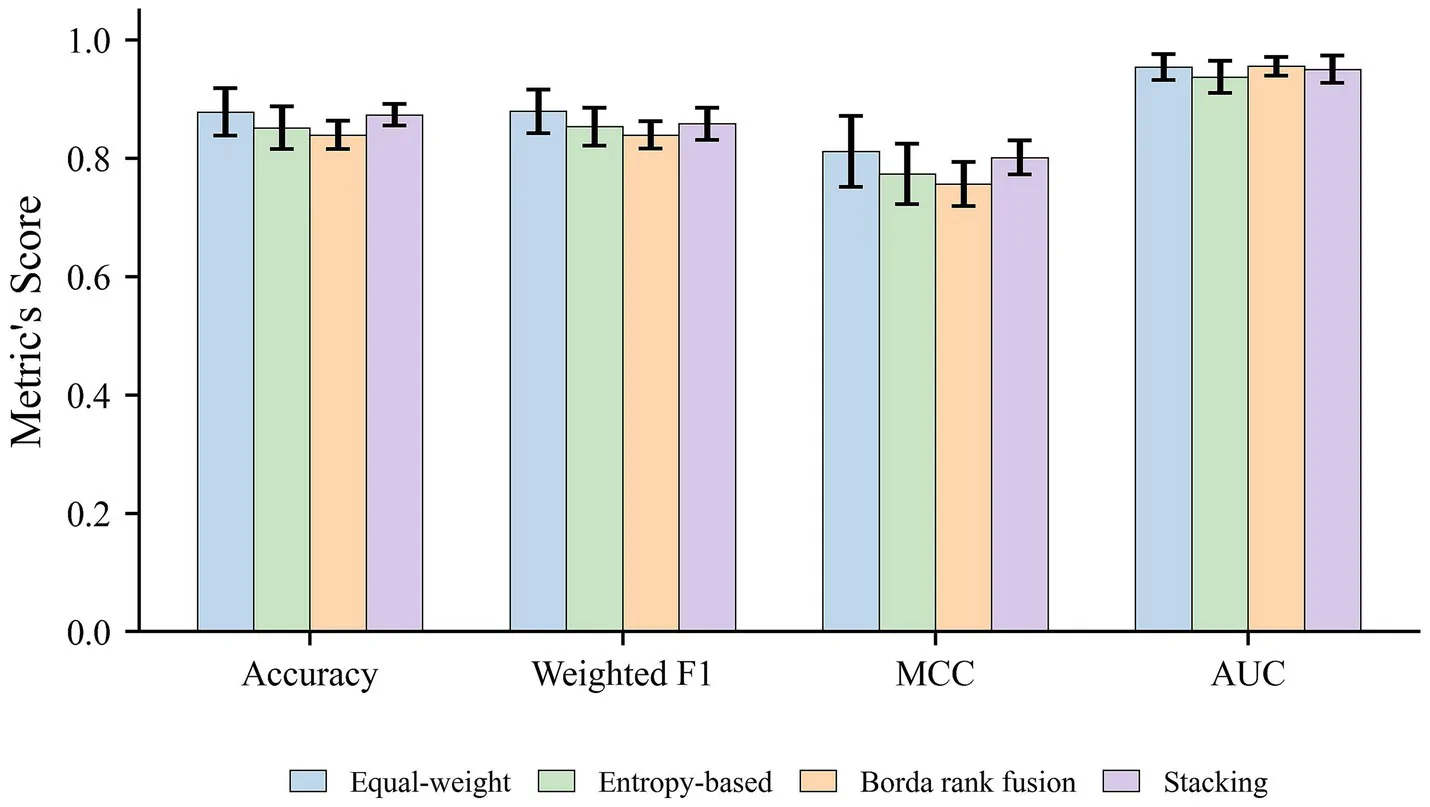
Comparison of different decision-level fusion strategies, across accuracy, weighted F1-score, MCC, and AUC.

**Figure 7:**
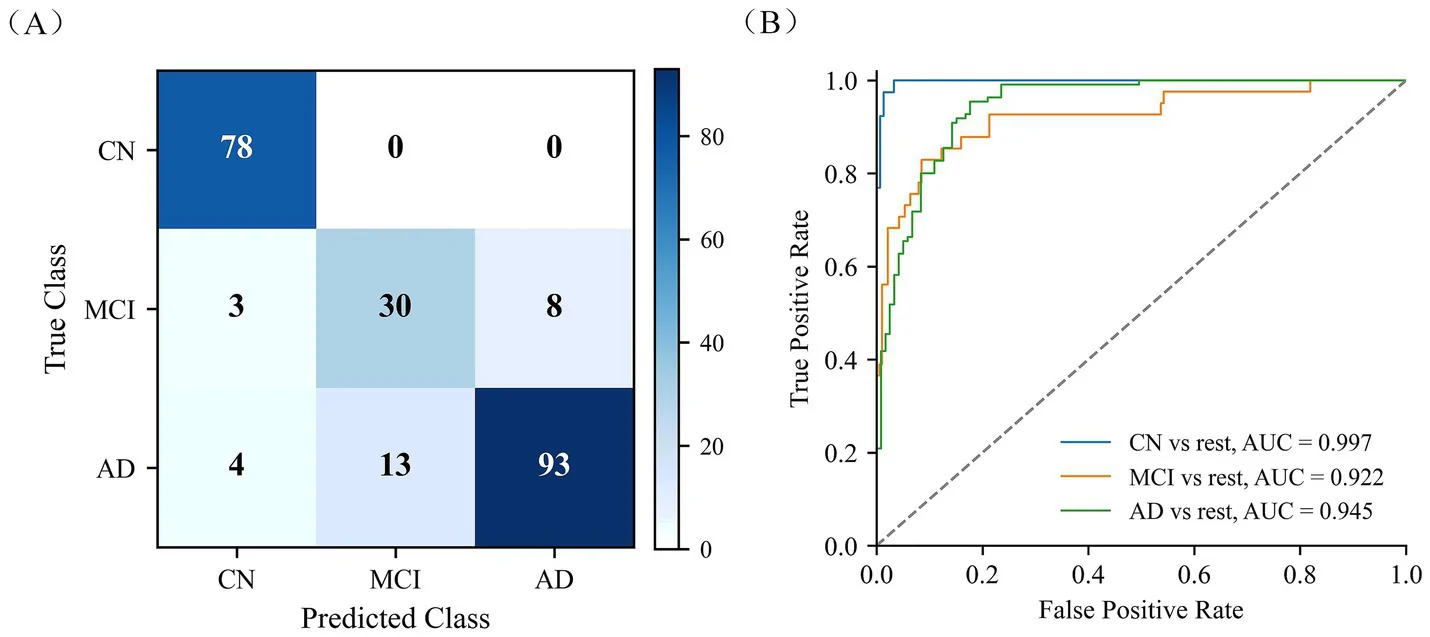
Confusion matrix and ROC analysis of MEAF in the internal test set. **(A)** Confusion matrix for CN, MCI, and AD classification. **(B)** One-vs-rest ROC curves for each diagnostic category, with AUC for CN, MCI, and AD, respectively.

To further assess the effect of fusion strategy selection, different decision-level fusion methods were compared under the same experimental settings. Equal-weighted probability fusion achieved the best overall performance among the evaluated strategies, supporting its use as the final fusion approach in MEAF (Figure 6). The proposed MEAF framework, which integrates the imaging and clinical models, achieved a precision of 0.880, an F1-score of 0.877, an accuracy of 0.878, a specificity of 0.940, and an AUC of 0.955, outperforming the individual single-modality models (Table 2). Statistical comparisons using two-sided paired permutation tests showed that MEAF achieved significantly higher accuracy than both SAR-Net and XGBoost (both *p* < 0.05). In addition, class-wise performance metrics for CN, MCI, and AD, are provided in Supplementary Table S1. To further evaluate the classification performance of MEAF for AD cognitive staging, we compared it with two representative image-tabular fusion methods, including DAFT and HyperFusion (54, 55), under the same internal testing setting (Table 2). The confusion matrix further illustrated the class-specific prediction distribution of MEAF across CN, MCI, and AD categories, while the one-vs-rest ROC curves showed favorable discriminative performance for each diagnostic category (Figure 7). In the independent AIBL external validation cohort, MEAF retained reasonable classification, with a precision of 0.843, an F1-score of 0.803, an accuracy of 0.817, a specificity of 0.853, and an AUC of 0.834 (Table 2).

### 3.4 Subgroup analysis results

As illustrated in Table 3, consistent classification performance is demonstrated by MEAF across demographic subgroups. Except for the postgraduate-and-above education group, all subgroups achieved accuracies above 0.82. Across the demographic categories, the highest accuracy was observed in the university education group with 0.934 (95% CI: 0.882–0.987), and the 66 to 80-year age group, with 0.901(95% CI: 0.851–0.950). Relatively lower performance was observed in the postgraduate-and-above group with 0.774 (95% CI: 0.660–0.887) and the greater than 80-year group with 0.822 (95% CI: 0.711–0.933). In terms of sex, slightly higher classification accuracy was observed in males than in females, with 0.887 (95% CI: 0.823–0.944) versus 0.867 (95% CI: 0.800–0.924). Pairwise permutation tests showed generally comparable performance across age, sex, and education subgroups. After Benjamini–Hochberg FDR correction, only the weighted F1-score differed significantly between the university and postgraduate-and-above education groups. Detailed results are provided in Supplementary Table S2.

**Table 3:** Subgroup performance of MEAF across age, sex, and education groups.

Characteristic	Subgroup	Accuracy (95% CI)	F1-score (95% CI)	MCC (95% CI)
Age	≤65	0.860 (0.767–0.953)	0.855 (0.742–0.953)	0.795 (0.654–0.929)
	66–80	0.901 (0.851–0.950)	0.899 (0.842–0.949)	0.835 (0.753–0.913)
	>80	0.822 (0.711–0.933)	0.824 (0.707–0.934)	0.714 (0.543–0.886)
Education	Below high school	0.900 (0.750–1.000)	0.945 (0.850–1.000)	0.834 (0.645–1.000)
	High school	0.901 (0.831–0.958)	0.895 (0.813–0.961)	0.821 (0.697–0.927)
	University	0.934 (0.882–0.987)	0.933 (0.873–0.987)	0.899 (0.814–0.980)
	Postgraduate and above	0.774 (0.660–0.887)	0.767 (0.646–0.881)	0.677 (0.515–0.825)
Sex	Female	0.867 (0.800–0.924)	0.869 (0.803–0.925)	0.794 (0.692–0.884)
	Male	0.887 (0.823–0.944)	0.883 (0.816–0.943)	0.815 (0.717–0.904)

### 3.5 Interpretability analysis results

**Figure 8:**
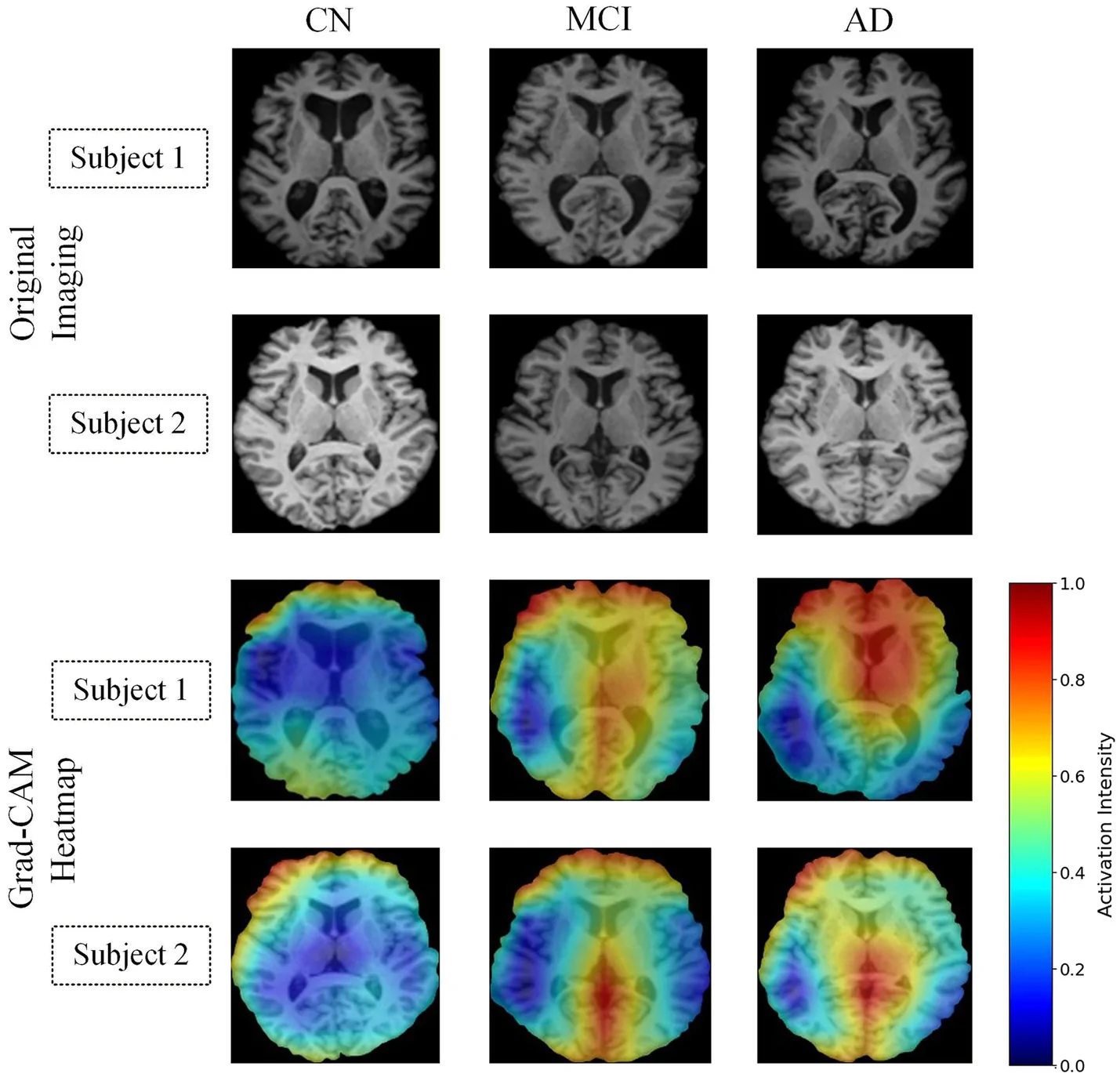
Grad-CAM visualizations for MRI models. Each column presents a representative subject from the CN, MCI, and AD groups, respectively. Color gradients from blue to red indicate varying degrees of activation intensity, with red signifying regions of highest predictive importance for AD risk.

Grad-CAM heatmaps were generated using a color-coding scheme in which red regions indicated areas exerting the greatest influence on model predictions, whereas blue regions indicated areas of lesser influence (56). As illustrated in Figure 8, MCI subjects exhibited prominent activation predominantly in the frontal and precentral gyri, reflecting early-stage structural changes. In contrast, AD subjects exhibited extensive activation in memory- and cognition-related regions, including the hippocampus, thalamus, and frontal lobe, consistent with disease progression along well-established neurodegenerative trajectories.

**Figure 9:**
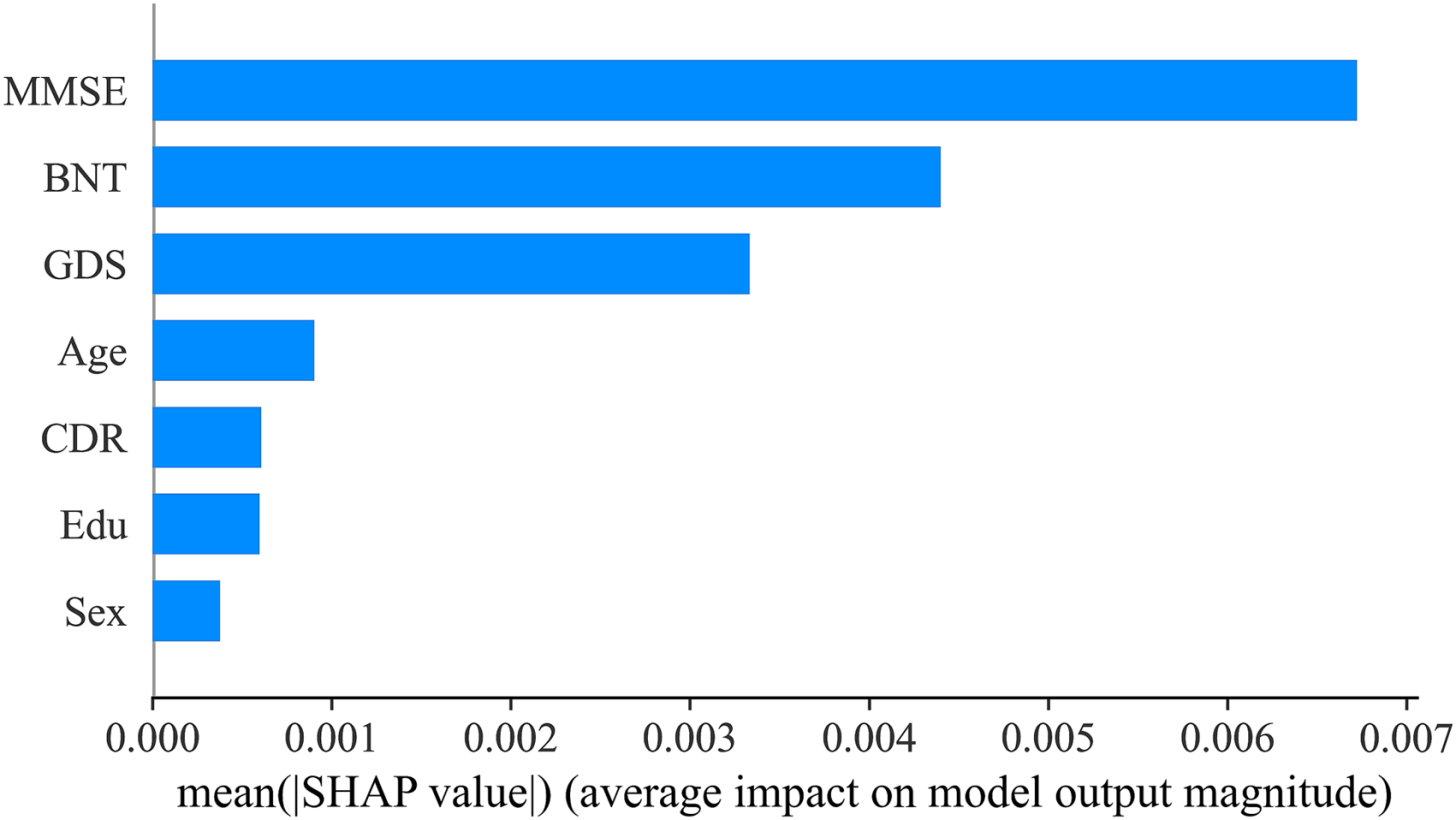
Clinical feature importance derived from SHAP analysis.

Furthermore, as shown in Figure 9, cognitive assessment indicators including MMSE, BNT and GDS occupy relatively high feature importance in the clinical module, which matches the comprehensive evaluation logic adopted in routine cognitive consultation. The Top-5 SHAP-ranked features were highly stable across bootstrap iterations, and removing top-ranked features progressively reduced model performance. These findings support the stability and predictive relevance of the SHAP-identified clinical features, with detailed results provided in Supplementary Tables S3, S4.

## 4 Discussion

In this study, we developed MEAF, a modular multimodal ensemble framework for differential classification across the AD cognitive spectrum. By integrating MRI-based and clinical-model predictions at the decision level, MEAF achieved favorable performance in the internal test cohort and maintained reasonable performance in the independent external validation cohort, providing preliminary support for its external generalizability. The framework exhibits stable performance across demographic subgroups, thereby supporting its broad applicability. The framework employs decision-level fusion strategies that offer multiple advantages, including effective management of heterogeneous data sources, dimensionality reduction, noise suppression, enhanced predictive performance, and improved interpretability (28, 57). Consistent with previous multimodal AD studies, our findings support the complementary diagnostic value of clinical and imaging information (29–31). Unlike tightly coupled feature-level fusion approaches, MEAF integrates modality-specific predictions at the decision level, preserving model modularity and modality-specific interpretability. Therefore, MEAF provides a complementary, clinically oriented framework that emphasizes modular flexibility and modality-specific interpretability.

The datasets utilized in this study include multiple sources, scanner vendors, and variable scanning conditions, including different magnetic field strengths. This diversity supports the robustness of the framework across devices and cohorts and underscores its clinical applicability (28). Taken together, the t-SNE, silhouette, and domain-classifier results provide complementary evidence of limited domain-related separability in the preprocessed MRI features, although residual domain effects cannot be fully excluded. Future studies should further evaluate and mitigate such effects through more advanced domain harmonization and adaptation strategies. While existing models effectively leverage MRI and clinical features for multimodal classification, most approaches are limited to binary categories such as CN versus AD, and therefore failing to capture the complexity inherent in real-world clinical decision-making (58). To address this limitation, a memory clinic workflow was simulated that covers the full cognitive spectrum, thereby transforming the predictive model into a clinically applicable decision-support tool.

Although the proposed model demonstrates strong generalizability across demographic subgroups, a certain degree of performance heterogeneity is still observed. In the age-stratified analysis, the 66–80-year age group achieved the highest accuracy (0.901), whereas a relatively lower accuracy was observed in the over-80-year group (0.822). This difference may be associated with a higher prevalence of multimorbidity and mixed pathological changes in the oldest population. It may also be attributed to the greater likelihood of complex or atypical clinical presentations in this group, which may reduce the ability to discriminate disease related features. In the education-level subgroup, the postgraduate-and-above group showed the lowest accuracy (0.774). This finding is partly consistent with the cognitive reserve hypothesis, which suggests that individuals with higher educational attainment can tolerate greater levels of neuropathological burden while maintaining relatively preserved clinical function, thereby making early disease identification more challenging (59). The subgroup findings should be interpreted cautiously because some education subgroups had relatively limited sample sizes and potential class imbalance, which may affect the stability of pairwise comparisons. Overall, these findings highlight the importance of considering demographic heterogeneity in clinical applications.

The need for transparent and interpretable AI models has also been highlighted in broader neurological applications, including fuzzy logic-based approaches for neurological signal analysis (60). Owing to the “black-box” nature of AI models, clinicians may be reluctant to rely fully on these models for clinical decision-making (61). The performance of the proposed predictive model is supported by established clinical indicators and well-validated imaging progression patterns associated with AD. Structural atrophy in specific brain regions is widely recognized as being closely associated with disease onset (62). Grad-CAM heatmaps derived from the imaging model reveal pronounced activations in the hippocampus and frontal lobes, regions widely recognized as hallmarks of cognitive impairment (63–65). Similarly, SHAP analysis allows the clinical branch of the model to be interpreted in the context of established cognitive assessment frameworks by quantifying the contributions of individual features, such as MMSE (66). Overall, the interpretability of both models links data-driven predictions to established clinical evidence, thereby enhancing their credibility and acceptability in clinical practice.

Several limitations of this study should be acknowledged. Models trained primarily on AD-specific features may fail to capture the complex phenotypes of mixed dementia, including concurrent AD and Lewy body dementia (67), highlighting the need for multi-label or mixed-dementia classification frameworks in future research. In addition, MEAF was constructed using established algorithms rather than a newly designed backbone network. Future work should explore task-specific architectures or end-to-end multimodal learning mechanisms to further improve methodological innovation and model adaptability. Although an independent AIBL external validation cohort was included, several clinical variables, including Edu, GDS, and BNT, were unavailable in AIBL, as detailed in Table 1, which may have affected the performance of the clinical and fusion models. Future studies with larger external cohorts and harmonized imaging, clinical, and biomarker data are needed to further validate MEAF. Moreover, Grad-CAM provides qualitative visual interpretation rather than quantitative anatomical verification. Future studies should incorporate ROI overlap analysis and expert neuroimaging review to further validate the anatomical consistency of model explanations. Finally, although MEAF showed promising performance in retrospective multicenter datasets, its clinical applicability requires further evaluation in prospective studies. Future work should incorporate clinician-in-the-loop assessment and real-world workflow testing to further evaluate how MEAF may support multimodal cognitive staging in practical clinical settings.

## 5 Conclusion

In conclusion, we developed and retrospectively evaluated an interpretable multimodal fusion framework for classifying cognitive stages across the AD continuum using routinely collected clinical and imaging data. The framework showed favorable classification performance across retrospective multicenter datasets, while the imaging regions and clinical variables identified in the interpretability analyses were broadly consistent with previously reported disease-related patterns. These findings provide preliminary evidence supporting further evaluation of the framework. Prospective validation, clinician-in-the-loop assessment, and real-world workflow studies are required before its potential clinical utility can be established.

## Data Availability

Publicly available datasets were analyzed in this study. Four datasets were analyzed in this study: ADNI: Repository: LONI Image and Data Archive, URL: https://adni.loni.usc.edu/, Accession number: sa000002; OASIS: Repository: OASIS Brains, URL: https://www.oasis-brains.org/, no accession number; NACC: Repository: National Alzheimer’s Coordinating Center, URL: https://naccdata.org/, no accession number; AIBL: Repository: Australian Imaging, Biomarkers and Lifestyle Flagship Study of Ageing, URL: https://www.aibl.csiro.au/, no accession number.
