# Supplementary material for: An interpretable multimodal ensemble assessment framework for Alzheimer’s disease cognitive staging
Source: Front Neurol. 2026 Aug 5;17:1911900. doi: 10.3389/fneur.2026.1911900 (PMC13488312; doi:10.3389/fneur.2026.1911900)
Supplement: Supplementary file 1 [file Supplementary_file_1.DOCX]

**Supplementary Table S1.** Class-wise performance of the imaging, clinical, and fusion models for three-class classification of CN, MCI, and AD.

| Model | Class | SEN | PRE | SPC | ACC | F1-score | AUC |
| --- | --- | --- | --- | --- | --- | --- | --- |
| SAR-Net | CN | 1.00 | 0.907 | 0.947 | 0.965 | 0.951 | 0.995 |
|  | MCI | 0.683 | 0.596 | 0.899 | 0.860 | 0.636 | 0.895 |
|  | AD | 0.782 | 0.896 | 0.916 | 0.852 | 0.835 | 0.922 |
| XGBoost | CN | 0.821 | 0.744 | 0.854 | 0.843 | 0.780 | 0.927 |
|  | MCI | 0.683 | 0.824 | 0.968 | 0.917 | 0.747 | 0.958 |
|  | AD | 0.845 | 0.853 | 0.866 | 0.856 | 0.849 | 0.921 |
| MEAF | CN | 1.000 | 0.918 | 0.954 | 0.969 | 0.957 | 0.997 |
|  | MCI | 0.732 | 0.698 | 0.931 | 0.895 | 0.714 | 0.922 |
|  | AD | 0.845 | 0.921 | 0.933 | 0.891 | 0.882 | 0.945 |

**Supplementary Table S2.** Pairwise significance analysis of subgroup performance.

| Characteristic | Subgroup | Accuracy  (adjusted *P*) | F1-score  (adjusted *P*) | MCC  (adjusted *P*) |
| --- | --- | --- | --- | --- |
| Age | ≤65 vs 66-80 | 0.774 | 0.662 | 0.611 |
|  | ≤65 vs >80 | 0.774 | 0.758 | 0.611 |
|  | 66-80 vs >80 | 0.547 | 0.526 | 0.509 |
| Education | Below high school vs High school | 1.000 | 0.716 | 0.859 |
|  | Below high school vs University | 0.765 | 0.993 | 0.476 |
|  | Below high school vs Postgraduate and above | 0.655 | 0.214 | 0.455 |
|  | High school vs University | 0.765 | 0.601 | 0.455 |
|  | High school vs Postgraduate and above | 0.226 | 0.188 | 0.455 |
|  | University vs Postgraduate and above | 0.067 | 0.028 | 0.102 |
| Sex | Female vs Male | 0.686 | 0.770 | 0.767 |

Note: P values were obtained using two-sided pairwise permutation tests and adjusted for multiple comparisons using the Benjamini–Hochberg false discovery rate procedure within each subgroup characteristic and performance metric. An FDR-adjusted P value < 0.05 was considered statistically significant.

**Supplementary Table S3.** Bootstrap-based stability analysis of SHAP feature rankings.

| Feature | Top-5 frequency | Top-5 frequency ratio |
| --- | --- | --- |
| MMSE | 100 | 1.00 |
| BNT | 100 | 1.00 |
| GDS | 100 | 1.00 |
| Age | 100 | 1.00 |
| CDR | 87 | 0.87 |
| Edu | 10 | 0.10 |
| Sex | 3 | 0.03 |

**Supplementary Table S4.** SHAP-guided feature ablation analysis.

| Setting | Number of features | ΔAUC | ΔMCC |
| --- | --- | --- | --- |
| All features | 7 | 0.000 | 0.000 |
| Remove Top-1 SHAP features | 6 | -0.054 | -0.216 |
| Remove Top-3 SHAP features | 4 | -0.224 | -0.444 |
| Remove Top-5 SHAP features | 2 | -0.344 | -0.633 |
| Only Top-5 SHAP features | 5 | -0.004 | -0.007 |
